# Effect of Latitude on Seasonality of Tuberculosis, Australia, 2002–2011

**DOI:** 10.3201/eid1811.120456

**Published:** 2012-11

**Authors:** Jennifer H. MacLachlan, Caroline J. Lavender, Benjamin C. Cowie

**Affiliations:** Victorian Infectious Diseases Reference Laboratory, North Melbourne, Victoria, Australia (J.H. MacLachlan, C.J. Lavender, B.C. Cowie);; Victorian Infectious Diseases Service, Parkville, Victoria, Australia (B.C. Cowie);; and University of Melbourne, Parkville (B.C. Cowie)

**Keywords:** tuberculosis, epidemiology, public health, surveillance, seasonal variation, latitude, periodicity, vitamin D, sunlight, migration, Australia, Mycobacterium tuberculosis and other mycobacteria

## Abstract

Seasonal variation in tuberculosis diagnoses recently has been reported in various populations. In Australia, seasonality of tuberculosis diagnoses was more pronounced in areas where UV exposure is reduced and vitamin D deficiency is more prevalent. Our findings suggest vitamin D deficiency as a factor in disease activation.

Tuberculosis profoundly affects human health, with 5.7 million new or recurrent cases reported and >1 million deaths attributed to the infection by the World Health Organization in 2010 (*1*). Tuberculosis has afflicted humans for millennia, and the potential of sunlight in prevention and treatment has been recognized for more than a century. Before the introduction of antimycobacterial therapy, several therapeutic approaches for tuberculosis were attempted. One example was creation of sanatoria, a positive aspect of which was thought to have been exposure to sunlight:

The ultraviolet rays are absorbed and are beneficial to the general health, even counterbalancing a deficiency in vitamins, to some extent. They have been… used as a treatment for tuberculosis of bones and joints, in which they seem to have a direct effect on the bacteria (*2*).

The nexus between exposure to sunlight and risk for active tuberculosis has been increasingly recognized, with a putative mechanism being vitamin D deficiency that reduces the ability of macrophages to kill intracellular *Mycobacterium tuberculosis* (*3*). Vitamin D deficiency has been associated with latent (*4*) and active (*5*) tuberculosis, and seasonality in the number of tuberculosis diagnoses has recently been reported in several regions (*3*,*6*).

Australia extends from latitudes 10° to 44° south, from equatorial to temperate climatic zones (Figure 1); as a consequence, incident sunlight intensity and vitamin D synthesis vary widely (*8*). We hypothesized that active tuberculosis cases diagnosed in Australia would display seasonality in regions further from the equator but less so in the tropics and that this seasonality would manifest as increased tuberculosis activity several months after nadir sunlight levels.

**Figure 1:**
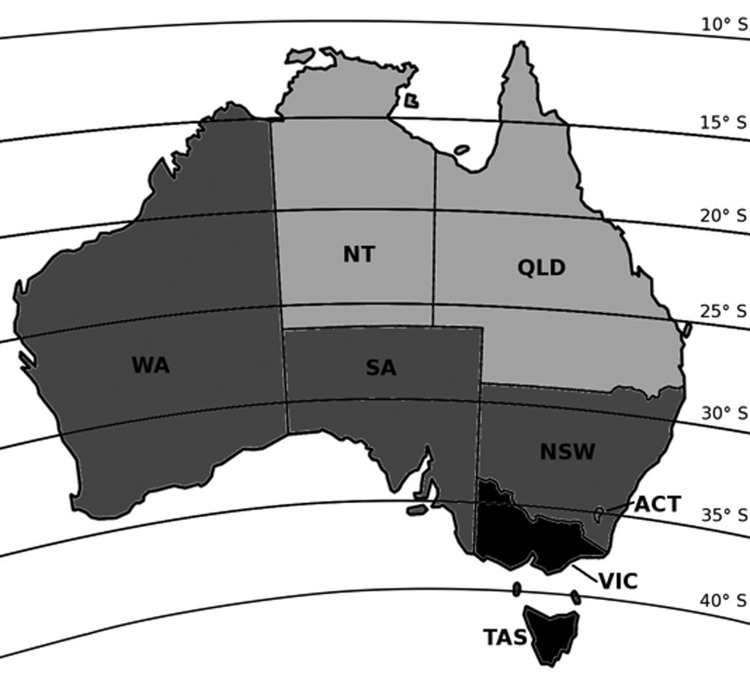
Australia with latitude lines, divided into north, central, and south regions according to latitude and ultraviolet (UV) exposure. Although Western Australia extends to the tropics, >90% of the state’s population lives below latitude 30°S (*7*). ACT, Australian Capital Territory; NSW, New South Wales; NT, Northern Territory; QLD, Queensland; SA, South Australia; TAS, Tasmania; VIC, Victoria; WA, Western Australia. Black, south region; dark gray, central region; light gray, north region.

Tuberculosis is a nationally notifiable disease in Australia, with medical practitioners and testing laboratories legally required to report all tuberculosis cases in all Australian states and territories. Each jurisdiction reports all confirmed tuberculosis cases to the National Notifiable Diseases Surveillance System, managed by the Australian Government Department of Health and Ageing (*9*). Confirmed tuberculosis cases are classified as either new or relapsed at the time of infection, consistent with World Health Organization case notification definitions (*1*,*9*).

## The Study

We obtained all notifications of tuberculosis to the National Notifiable Diseases Surveillance System during January 2002–December 2011. States and territories were divided on the basis of latitude into north, central, and south regions (Figure 1). Australia data for seasonal UV exposure (*10*,*11*) and vitamin D levels (*8*,*11*) in these regions were obtained and compared with tuberculosis notifications to identify potential patterns in incidence according to these changing environmental factors. We derived the number of tuberculosis notifications per 100,000 population using midpoint population denominator estimates for each state taken from the 2006 census (*7*).

Seasonality in tuberculosis notifications was assessed for the whole of Australia and individually for each region by using Edward’s test of seasonality, and the difference in magnitude of seasonality between regions was compared by using the amplitude of cyclic variation. The Wilcoxon rank-sum test was used to test the hypothesis that the mean tuberculosis notifications per 100,000 persons in the south did not differ from those in central states, separately for January–June and July–December.

**Figure 2:**
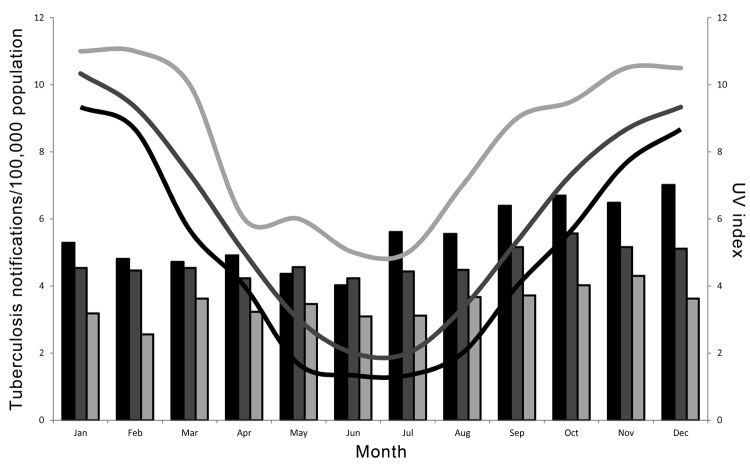
Tuberculosis notifications per 100,000 population (bars) and ultraviolet (UV) index (lines), Australia 2002–2011. Black, south region; dark gray, central region; light gray, north region.

During 2002–2011, a total of 11,576 tuberculosis cases were reported in Australia (Table), with an apparent increase in mean number of cases reported during September–December. This effect was more pronounced in temperate than in tropical areas (Figure 2). Notifications of tuberculosis began to rise in temperate areas in July and peaked in October in the central region and in December in the south. Furthermore, incidence of tuberculosis diagnosis per 100,000 population was similar in the central and south regions in the first half of the calendar year (p = 0.20) but diverged significantly for July–December (p = 0.006).

**Table:** Number and rate* of tuberculosis notifications, Australia, 2002–2011

Month	South			Central			North			Total	
	Mean cases (range)	Rate		Mean cases (range)	Rate		Mean cases (range)	Rate		Mean cases (range)	Rate
Jan	30.2 (25–37)	5.3		48.4 (35–62)	4.5		13.7 (8–20)	3.2		92.3 (73–103)	4.5
Feb	27.6 (21–37)	4.8		47.5 (38–58)	4.5		11 (5–14)	2.6		86.1 (74–105)	4.2
Mar	27.8 (17–40)	4.7		47.6 (36–56)	4.5		15.6 (8–24)	3.6		91.0 (66–109)	4.4
Apr	29.4 (18–41)	4.9		43.8 (21–65)	4.2		13.9 (6–20)	3.2		87.1 (54–114)	4.2
May	25.3 (10–35)	4.4		48.4 (40–58)	4.6		14.9 (10–29)	3.5		88.6 (74–104)	4.3
Jun	24 (16–39)	4.0		44.2 (35–56)	4.2		13.3 (8–26)	3.1		81.5 (62–98)	3.9
Jul	32.9 (26–49)	5.6		46.4 (36–65)	4.4		13.4 (7–24)	3.1		92.7 (73–110)	4.5
Aug	31.9 (18–42)	5.6		47.6 (34–57)	4.5		15.8 (10–26)	3.7		95.3 (72–121)	4.6
Sep	37.2 (27–44)	6.4		54.3 (45–70)	5.2		16 (8–26)	3.7		107.5 (85–135)	5.2
Oct	39.7 24–61)	6.7		57.9 (42–89)	5.6		17.3 (13–30)	4.0		114.9 (81–166)	5.5
Nov	38.1 (27–50)	6.5		53.9 (44–78)	5.2		18.5 (13–22)	4.3		110.5 (94–122)	5.3
Dec	41.6 (25–58)	7.0		52.9 (33–67)	5.1		15.6 (9–31)	3.6		110.1 (81–139)	5.3

*Per 100,000 population.

When sunlight exposure was taken into account, the fall in UV index <3 in the south and central regions was followed 2–3 months later by notable increases in tuberculosis diagnoses. In northern Australia, where the average UV index does not drop below 4 even during winter, the seasonal trend was less marked (Figure 2).

Formal tests of seasonality indicated that the number of tuberculosis notifications per 100,000 varied significantly by month for the whole of Australia and for each region (p<0.001). The effect was more pronounced in the south than in the central and north regions (amplitudes of cyclic variation 0.207, 0.094, and 0.101, respectively).

## Conclusions

The cyclical patterns in tuberculosis activity in this study have been observed in other settings (*3*,*12*), and a difference according to latitude has been found in India, with more seasonality in northern regions (*13*). No effect of latitude was found in a recent study of tuberculosis seasonality in the United States (*6*). This difference may be due to the relative latitudes of these locations; all latitudes of the continental United States are further from the equator (30°N–55°N) than are latitudes in India (8°N–37°N) and Australia (10°S–44°S).

The seasonal pattern according to latitude corresponds with findings about vitamin D deficiency by region in Australia in a national study of 11,247 adults (*8*), which found that the odds of vitamin D deficiency were 3–4 times higher in winter/spring than in summer and were more than double for persons residing in latitudes >35°S (Figure 1). Although ecologic studies, such as that presented here, cannot address questions of causation, the temporal lag between low UV exposure, vitamin D deficiency, and increased tuberculosis diagnoses supports the argument that vitamin D deficiency leads to tuberculosis, and not the converse.

Persons born overseas accounted for 86.4% of tuberculosis cases in Australia in 2007, despite constituting ≈30% of the population. Aboriginal and Torres Strait Islander peoples represented 23% of cases in Australia-born persons, despite constituting 3.7% of the Australian-born population (*7*). The predominant countries of birth of Australians born overseas and in whom tuberculosis was diagnosed during that year were (in descending order) India, Vietnam, the Philippines, People’s Republic of China, Indonesia, Papua New Guinea, Sudan, Myanmar, Nepal, Bangladesh, and Pakistan (*9*). In addition to the increased risk for exposure to tuberculosis early in life in persons born overseas, increased susceptibility to vitamin D deficiency also might increase the risk for reactivation of tuberculosis in these groups, with vitamin D deficiency more common among indigenous Australians (*14*) and migrants from high tuberculosis prevalence countries (*8*,*15*).

These findings should guide clinical and public health practice in Australia and similar countries that have high migration from tuberculosis-endemic populations and substantial risk for vitamin D deficiency. Clinicians should provide individualized advice to persons at higher risk for tuberculosis, especially those who have migrated from tuberculosis-endemic areas and those in whom latent tuberculosis already has been diagnosed, about vitamin D levels and consideration of supplementation as appropriate.
